# Tired minds, normal scores: rethinking cognitive fatigue in multiple sclerosis

**DOI:** 10.3389/fneur.2025.1664734

**Published:** 2025-10-08

**Authors:** Davide Spinetti, Ignazio Roberto Zarbo

**Affiliations:** ^1^Department of General Psychology (DPG), University of Padua, Padua, Italy; ^2^Neurology Unit, AOU Sassari, Sassari, Italy

**Keywords:** multiple sclerosis, cognitive fatigue, PASAT, Symbol Digit Modalities Test, Modified Fatigue Impact Scale (MFIS), Fatigue Scale for Motor and Cognitive Functions, ecological momentary assessment

## Abstract

Cognitive fatigue is among the most pervasive and disabling symptoms experienced by individuals with multiple sclerosis (MS), yet it remains underrecognized and undertreated in both research and clinical settings. Despite its prevalence, cognitive fatigue is often confused with general fatigue or overlooked in standard neuropsychological assessments that rely on brief, decontextualized performance measures. This perspective argues for a paradigmatic shift in how cognitive fatigue is conceptualized and managed. This perspective define it as a core, dynamic dysfunction affecting the cognitive system’s capacity to sustain effort over time, distinct from both general and subjective fatigue. A growing body of evidence, highlighted in this article, shows a frequent dissociation between perceived fatigue and objective fatigability, underscoring a fundamental flaw in current assessment tools. The authors call for the integration of prolonged, dynamic, and ecologically valid measures, such as extended neuropsychological tasks and digital phenotyping, into clinical protocols. We propose repositioning cognitive fatigue as a distinct clinical target, requiring its own specific strategies for identification, monitoring, and intervention. Reframing cognitive fatigue in this way offers a critical step toward more accurate diagnostics and truly individualized care, bridging the gap between clinical findings and the patient’s lived experience.

## Introduction

Multiple sclerosis (MS) is well known for its motor symptoms, sensory disturbances, and visual impairments, but perhaps less visible, though no less disabling, are the cognitive consequences of the disease (1, 2). Among these, cognitive fatigue stands out as both extremely common and poorly understood. Studies suggest that between 65 and 90% of individuals with MS report experiencing some form of fatigue, and a significant portion of them identify cognitive fatigue as the most debilitating aspect (3–5). Despite its prevalence and profound impact on quality of life, cognitive fatigue remains underrepresented in diagnostic frameworks and therapeutic approaches. A core challenge in addressing this symptom is its elusive nature and a lack of consistent terminology. For the purpose of this Perspective, we define “Fatigue” as a broad, overarching term for a state of weariness that can be physical, mental, or a combination of both (6) (see glossary). Within this, we distinguish between:

- Subjective cognitive fatigue: the patient’s self-reported, internal sensation of mental exhaustion or lack of energy for an ongoing cognitive task (5, 7, 8).
- Objective cognitive fatigability: a measurable decline in cognitive performance (e.g., speed, accuracy) over the duration of a sustained or repeated task (8, 9).

The distinction between the subjective feeling and the objective, measurable decline is crucial, as a growing body of evidence shows these two dimensions are often dissociated, with weak or no correlation between them (10, 11). This paradox highlights a fundamental failing of current assessment paradigms, which are primarily based on brief, decontextualized performance measures. Standard neuropsychological tests, while effective at identifying stable deficits, often fail to capture the dynamic and context-sensitive nature of cognitive fatigability, and neuropsychological tests alone may fail to provide clinical predictability (42). As a result, a patient may perform “within normal limits” on a standard cognitive battery yet struggle to maintain attention at work or follow a conversation for a prolonged period (12). This mismatch between clinical findings and lived experience underscores the urgent need for a new conceptual and methodological approach. This article argues for a paradigmatic shift in how cognitive fatigue in MS is conceptualized and managed. Rather than treating it as a vague, secondary complaint, the Authors propose to reposition it as a core, dynamic dysfunction that requires specific assessment tools, dedicated interventions, and centrality in clinical decision-making. This perspective aims to illuminate why current models fail, what a more ecologically valid approach would look like, and how shifting our clinical priorities might transform care for people with MS.

## The paradox of cognitive fatigue: when normal is not normal

Cognitive fatigue, as distinct from physical fatigue, refers to the gradual decline in cognitive efficiency and mental energy during sustained mental activity (7, 8). One of the most frustrating experiences reported by people with MS is the mismatch between how they feel and how they are clinically assessed (43). It is not merely a subjective complaint of tiredness, nor is it fully captured by momentary lapses in attention or performance scores. Rather, it reflects a more fundamental limitation in the brain’s ability to maintain cognitive performance over time. This degradation often emerges during prolonged tasks, multitasking environments, or even everyday conversations, situations that are rarely simulated in conventional clinical testing. A patient might perform within the normal range on standard cognitive tests, achieving acceptable scores on attention, working memory, or processing speed, yet still report to be unable to follow conversations, focus at work, or read more than a few pages of text (13). This dissonance is often interpreted, mistakenly, as exaggeration or emotional overlay. In reality, it reflects a fundamental failure of current testing paradigms to capture the temporal dynamics and ecological reality of cognitive fatigue (7, 14). What makes cognitive fatigue particularly insidious in MS is its elusive nature: it fluctuates across time and contexts, is influenced by psychological and environmental factors, and often coexists with depression, anxiety, or sleep disturbances (15, 16). It does not always correlate with disease progression, lesion load, or the presence overt cognitive deficits (17). The conceptual ambiguity surrounding cognitive fatigue has impeded its recognition as a core clinical concern. Recent research has begun to systematically address this gap by leveraging more dynamic paradigms and advanced neuroimaging, strengthening the argument that cognitive fatigue is a distinct and measurable entity.

Objective fatigability paradigms: beyond static, single-session scores, a growing body of literature uses objective paradigms to quantify cognitive fatigability. These methods measure a quantifiable performance decrement over time. Recent studies have successfully used prolonged or repeated blocks of the Symbol Digit Modalities Test (SDMT) and the Paced Auditory Serial Addition Test (PASAT) to reveal within-session declines in processing speed and attention that are not evident in a single, short administration (18, 19). Additionally, dual-task paradigms, which evaluate how simultaneous cognitive and motor demands affect performance (e.g., walking while performing serial subtractions), are increasingly used as an ecologically valid proxy for real-world multitasking and to reveal fatigability (20). Research shows that dual-task performance is significantly associated with deficits in processing speed and memory in people with MS (18, 21).

Dissociation of subjective vs. objective measures: the paradox between subjective and objective fatigue is a central theme in recent literature. Multiple studies have consistently shown weak or no correlation between patient-reported fatigue levels (e.g., using scales like the Modified Fatigue Impact Scale (MFIS)) and objective performance decrements (10, 11, 19). This dissociation highlights the critical need for a dual-pronged assessment that validates both the patient’s lived experience and a measurable decline in cognitive efficiency. A person’s feeling of being tired does not always predict their objective performance, and vice-versa, making both measures essential for a complete clinical picture. Finally, another important issue deserve to be highlighted: the self-report questionnaires currently present to measure and diagnose cognitive fatigue, they exclusively assess the subjective experience of fatigue in people with MS (5). These questionnaires are: Fatigue Severity Scale (FSS), the Fatigue Impact Scale (FIS), the Modified Fatigue Impact Scale (MFIS), the Fatigue Scale for Motor and Cognitive Functions (FSMC), or the Wuerzburg Fatigue Inventory for Multiple Sclerosis (WEIMuS).

Neural mechanisms: the underlying neurobiology of cognitive fatigue is a rapidly evolving field. While structural pathology (e.g., lesion load, atrophy) is often weakly correlated with subjective fatigue, recent neuroimaging studies point to a dysfunction in specific brain networks. A previous study has suggested how widespread axonal damage was associated to fatigue in MS (22). This has been associated with increased recruitment of brain resources, which may underlie patients’ subjective sense of effort during cognitive tasks. Research using fMRI has revealed altered functional connectivity in fronto-striatal and thalamocortical circuits in MS patients with fatigue, suggesting a failure of these networks to sustain efficient communication and resource allocation over time (23, 24). This network is called “fatigue circuit” (9, 25). Yet, structural pathology alone cannot explain the day-to-day variability of the symptom, nor its sensitivity to psychological factors such as anxiety, sleep disturbance, and stress (9). More recent evidence further highlights that decreased local functional connectivity within the basal nuclei is a key neural correlate of both subjective and objective fatigue in MS (26). This inconsistency suggests that contextual and person-specific variables, rather than brain damage alone, play a central role in modulating the fatigue experience (6). Thus, we face a paradox: cognitive fatigue is one of the most common and disabling symptoms in MS, yet it is both difficult to measure and often invisible in routine evaluations. It does not respect the boundaries of tests, nor does it conform to disease severity as measured by MRI or the EDSS. It is this very mismatch, between what is real to the patient and what is legible to the clinician, that demands a reappraisal of how we conceptualize, assess, and respond to cognitive fatigue.

Digital phenotyping and ecological momentary assessment (EMA): to bridge the gap between clinic-based findings and real-world functioning, a promising avenue is the use of digital tools and EMA. These methods, still nascent in MS, allow for the real-time tracking of cognitive performance and subjective state in daily life, capturing fluctuations and context-dependent factors that are difficult to observe in a clinical setting (16, 27, 28). This approach allows researchers and clinicians to move from a static to a dynamic, longitudinal understanding of the patient’s cognitive profile, establishing a real-time connection between daily symptoms and objective cognitive changes (18). Moreover, these types of emerging tools are underpinned by Artificial Intelligence, which may permit over time to collect an important number of data that permit to improve the disease management and patients outcomes (29, 30). In summary, this part has demonstrated that the dominant model of cognitive assessment, brief, static, decontextualized, fails to capture the dynamic and context-sensitive nature of cognitive fatigability. As a result, patients are left with an invisible but heavy burden: the sensation that thinking itself has become an exhausting activity.

## Reframing assessment: toward a dynamic, ecological neuropsychology

If cognitive fatigue is a dynamic process, our approach to assessment must be dynamic as well. What is needed is not merely the refinement of current tools, but a conceptual shift: from evaluating isolated cognitive capacities in static contexts to understanding how cognition holds up under prolonged, variable, and ecologically relevant conditions. Several researchers have advocated for such an approach. Sumowski et al. (24) argued that cognitive reserve, a person’s ability to maintain function despite brain pathology, depends not just on neuroanatomy but also on lifestyle, motivation, and environment. This concept is particularly relevant to fatigue, which may reveal itself only under the cognitive equivalent of “stress testing.” Rather than giving patients brief tasks with immediate feedback, we should be asking: how well does their cognition sustain over time, under conditions that simulate their real-life cognitive load?

### A multifaceted approach to assessment

Some preliminary models have tried to operationalize this. For example, performance decrement paradigms such as the PASAT have been used to assess cognitive fatigability by measuring changes in performance over time (31). Additionally, dual-task paradigms, which evaluate how simultaneous cognitive and motor demands affect performance, provide further insight into fatigue mechanisms in multiple sclerosis (20). Yet these are still relatively rare in clinical settings. Most patients with MS are not evaluated with prolonged tasks or multiple-session assessments, despite evidence that cognitive fatigability often only appears with repetition or over time (32). Another promising avenue is the integration of self-report tools with performance-based measures. Scales like the Modified Fatigue Impact Scale (MFIS) and the Fatigue Scale for Motor and Cognitive Functions (FSMC) provide useful insights into the subjective experience of fatigue, especially when tracked over time (33, 34). However, they are not designed to detect subtle performance decrements or fluctuations. Combining these with adaptive cognitive testing, ideally performed across longer time windows or even in daily life contexts, could yield a much richer understanding of a patient’s functional status. Moreover, ecological momentary assessment (EMA) and digital health tools are increasingly being used in other neurological populations to capture real-time fluctuations in cognitive state (28). While our goal here is not to advocate for technology per se, these methods underscore a broader point: cognition does not happen in a vacuum, and neither should its evaluation. A fatigue-aware neuropsychology must be one that respects variability, context, and the lived experience of the person being tested.

### A clinic-ready protocol

To operationalize this approach, We propose a clinical protocol that integrates these components into a step-by-step framework (Table 1). This protocol positions cognitive fatigue not as a secondary complaint, but as a primary area of concern, a new measurement dimension in test batteries and an independent target in clinical decision-making.

**Table 1:** Multi-faceted protocol for dynamic and ecological assessment of cognitive fatigability in multiple sclerosis.

Protocol step	Measurement domain	Example measures	Rationale
Step 1: fundamental assessment	Short standardized cognitive tests	SDMT, PASAT, Memory tests	To identify baseline cognitive function and focal deficits.
Step 2: subjective and impact assessment	Self-report scales	Modified Fatigue Impact Scale (MFIS), Fatigue Scale for Motor and Cognitive subscale	To capture the patient’s lived experience, their perception of fatigue, and its impact on daily life
Step 3: objective fatigability	Prolonged or repeated tasks	SDMT repeated, PASAT prolonged or repeated	To measure the decline in processing speed and attention with sustained effort, providing a quantitative index of fatigability
Step 4: ecological and multifactorial assessment	Dual-task paradigm and emerging digital tools	Dual-task paradigm (e.g., walking while doing serial subtractions), digital tools/EMA (e.g., smartphone apps)	To evaluate cognitive performance and the efficiency of resource allocation under stress, tracking real time fluctuations and context-dependent factors

## Clinical blind spots: why fatigue remains an afterthought

Despite its frequency and impact, cognitive fatigue rarely receives dedicated attention in routine clinical care. Most comprehensive neuropsychological evaluations in MS focus on standard cognitive domains, such as processing speed, memory, and executive function, without a systematic framework for evaluating fatigability. Clinicians may note a patient’s complaint of “mental exhaustion” but often interpret it as either nonspecific or a secondary symptom of mood disturbance. As a result, fatigue tends to be “delegitimized,” its clinical significance diminished by both its subjectivity and its mismatch with objective findings (Table 2).

**Table 2:** Glossary of key words.

Term	Definition
Subjective cognitive fatigue	Self-reported sensation of mental exhaustion or lack of cognitive energy.
Objective cognitive fatigability	Observable decline in cognitive performance (e.g., accuracy, speed) during sustained or repeated tasks.
Dual-task cost	Performance decrement when a person performs two tasks simultaneously (e.g., walking+mental arithmetic), reflecting cognitive-motor interference.
Decrement paradigm	Experimental design that measures within-session performance decline by repeating the same cognitive task across multiple blocks.
Ecological Momentary Assessment (EMA)	Repeated, real-time self-report or cognitive testing in daily life, often delivered through smartphones or wearable devices, to capture fluctuations in fatigue.
Digital phenotyping	Use of digital tools (apps, sensors, wearables) to continuously track cognitive and behavioral states in real-world environments.

Several structural reasons contribute to this oversight.

1. The inadequacy of static assessment tools. Clinical neuropsychology remains anchored in a deficit model that privileges stable, measurable losses in function over dynamic impairments like fatigability. Brief tests are more practical in time-limited settings, and results that conform to standardized norms are easier to interpret and document. The limitations of current measurement methods may cause clinicians to experience difficulties in identifying and managing cognitive fatigue (Xavier (35)). For example, while the Multiple Sclerosis Functional Composite (MSFC) includes tests like the SDMT to assess processing speed, it does not address how performance changes over time or under stress (36, 37).
2. Inconsistent management guidelines. Guidelines for MS management often mention fatigue only in passing, and rarely differentiate between physical and cognitive components. Furthermore, fatigue management strategies tend to focus on pharmacological interventions (e.g., amantadine, modafinil), despite limited and inconsistent evidence of benefit for cognitive fatigue specifically (38, 39).
3. The conflation with mood symptoms. There remains a pervasive tendency to equate cognitive fatigue with mood. While depression and anxiety can certainly exacerbate fatigue, and vice versa, they are distinct phenomena with different neurobiological underpinnings and treatment trajectories. Fatigue can persist in the absence of mood symptoms, and conversely, affective improvement does not necessarily resolve cognitive exhaustion (40, 44). Obscuring these boundaries risks both under-treatment and mismanagement.
4. The hidden disability. Ironically, patients often adapt their lives around cognitive fatigue long before clinicians recognize it. They reduce their working hours, avoid social interactions, or limit cognitively demanding tasks, not because of cognitive decline per se, but because of the mental cost of sustaining attention and effort (41). In this way, fatigue becomes a hidden cause of disability, reducing independence in ways that are hard to measure or recognize. In short, cognitive fatigue remains in the shadows of clinical practice not because it is rare or unimportant, but because it does not fit neatly into our existing categories. A shift is needed: from seeing fatigue as a secondary complaint to recognizing it as a primary domain of dysfunction deserving structured assessment and intervention.

## Conclusion

Cognitive fatigue in multiple sclerosis is not a vague or secondary symptom. It is a core manifestation of the disease, one that undermines daily functioning, self-efficacy, and quality of life, even in the absence of overt cognitive decline. And yet, it remains invisible to many of the tools and frameworks we rely on for diagnosis and care. This Perspective has argued that cognitive fatigue deserves a central place in the neuropsychological and clinical management of MS. Its elusive nature (fluctuating, multifactorial, and context-sensitive) demands an approach that is equally nuanced. Our proposed paradigmatic shift involves a dual-pronged re-evaluation. First, cognitive fatigue must be established as a new measurement dimension in our neuropsychological batteries, utilizing prolonged tasks and dynamic protocols to capture its temporal nature. Second, it must be recognized as an independent target in clinical decision-making, with specific interventions tailored to address mental endurance and sustainability. This requires not just better tools, but a rethinking of our clinical priorities: placing lived experience on equal footing with test performance. Importantly, cognitive fatigue should not be reduced to a measurement problem or a methodological inconvenience. It is a clinical reality that shapes how patients engage with the world, make decisions, and maintain autonomy. Recognizing and addressing cognitive fatigue is not only a scientific and clinical necessity but also an ethical imperative. This imperative is grounded in the principles of patient autonomy (supporting patients’ right to have their invisible symptoms acknowledged), equity in health (ensuring equal access to recognition and management of cognitive symptoms), and clinical responsibility (providing adequate care for what is measurable and meaningful in patients’ lives).

## Data Availability

The original contributions presented in the study are included in the article/supplementary material, further inquiries can be directed to the corresponding author.
